# Selectivity in glycosaminoglycan binding dictates the distribution and diffusion of fibroblast growth factors in the pericellular matrix

**DOI:** 10.1098/rsob.150277

**Published:** 2016-03-23

**Authors:** Changye Sun, Marco Marcello, Yong Li, David Mason, Raphaël Lévy, David G. Fernig

**Affiliations:** 1 Department of Biochemistry, Institute of Integrative Biology, University of Liverpool, Liverpool L69 7ZB, UK; 2 Centre for Cell Imaging, Institute of Integrative Biology, University of Liverpool, Liverpool L69 7ZB, UK

**Keywords:** glycosaminoglycan, heparan sulfate, fibroblast growth factor, extracellular matrix, growth factor diffusion

## Abstract

The range of biological outcomes generated by many signalling proteins in development and homeostasis is increased by their interactions with glycosaminoglycans, particularly heparan sulfate (HS). This interaction controls the localization and movement of these signalling proteins, but whether such control depends on the specificity of the interactions is not known. We used five fibroblast growth factors with an N-terminal HaloTag (Halo-FGFs) for fluorescent labelling, with well-characterized and distinct HS-binding properties, and measured their binding and diffusion in pericellular matrix of fixed rat mammary 27 fibroblasts. Halo-FGF1, Halo-FGF2 and Halo-FGF6 bound to HS, whereas Halo-FGF10 also interacted with chondroitin sulfate/dermatan sulfate, and FGF20 did not bind detectably. The distribution of bound FGFs in the pericellular matrix was not homogeneous, and for FGF10 exhibited striking clusters. Fluorescence recovery after photobleaching showed that FGF2 and FGF6 diffused faster, whereas FGF1 diffused more slowly, and FGF10 was immobile. The results demonstrate that the specificity of the interactions of proteins with glycosaminoglycans controls their binding and diffusion. Moreover, cells regulate the spatial distribution of different protein-binding sites in glycosaminoglycans independently of each other, implying that the extracellular matrix has long-range structure.

## 1. Background

The extracellular matrix has a central role in mediating communication between animal cells through mechanisms mediated by mechanical forces and soluble effectors. A large proportion of the soluble effectors, morphogens, growth factors, cytokines and chemokines that regulate animal development and homeostasis interact with glycosaminoglycans, particularly heparan sulfate (HS), of the extracellular matrix [1,2]. These interactions have been shown to exhibit varying degrees of specificity and selectivity at the tissue and at the molecular levels, and in a number of cases have been demonstrated to control the effectors' transport and intracellular signalling.

The glycosaminoglycans HS, chondroitin sulfate and dermatan sulfate are linear, sulfated polysaccharides covalently attached to core proteins to form proteoglycans. These are either associated with the cell membrane and resident in pericellular matrix or secreted, so resident in extracellular matrix. The long chains of HS (approx. 25–100 disaccharide units) consist of repeats of a disaccharide: d-glucosamine β 1–4 glucuronic acid or its epimer iduronic acid. The mature chains have a distinct domain structure of sequential blocks of unmodified disaccharides of *N*-acetyl glucosamine β 1–4 glucuronic acid, transition domains where *N*-acetyl glucosamine-containing disaccharides alternate with *N*-sulfated ones, and sulfated domains, where every glucosamine is *N*-sulfated and may also be *O*-sulfated on C_3_ and C_6_, and the uronic acid is often epimerized to iduronate, which may be 2-*O*-sulfated [1–3].

At least 435 extracellular regulatory proteins bind to transition and S-domains [4] (reviewed in [1,2]). At the molecular level, analysis of the structural basis of the interaction of individual proteins with HS and model polysaccharides (derivatives of the related heparin) shows that there is a selectivity by proteins for particular patterns of sulfation [5–7]. At the tissue level, clear differences in the expression of sulfated sugar structures have been demonstrated, which impact on cell communication in development, homeostasis and disease [8–13].

One important functional consequence of proteins binding HS is its potential to control the movement of effectors between cells. Endothelial cell extracellular matrix, in the 1980s, was demonstrated to be capable of storing fibroblast growth factor (FGF) 2, which could then transfer to its cellular receptors to stimulate the cells [14]. Later, HS in extracellular matrix was shown to control the diffusion of FGF2 [15], which indicated that HS had the potential to shape FGF2 gradients (FGF2 being both a growth factor and a morphogen [16]). Subsequently, the binding of a number of morphogens to HS was shown to control their diffusion in contexts ranging from *Drosophila* to vertebrates [17–22]. However, this may not be universal [23–25]. Moreover, it is not clear whether it is the selectivity of an effector for particular structures in the polysaccharide or just non-selective ion-exchange protein–polysaccharide interactions [26] that are important in regulating the effector's diffusion. A related issue is that HS in extracellular matrix has been viewed as homogeneous, that is, there is no variation in the distribution of binding sites below the scale of tissue compartments. However, work with nanoparticle-labelled FGF2 demonstrated that the distribution of its binding sites in fibroblast pericellular matrix is heterogeneous and clustered from length scales of approximately 20 nm to 1 µm and above [27]. Recently, biophysical experiments have shown that some effectors that bind HS can cross-link the chains of the polysaccharide [28]. This suggests that HS chains in extracellular matrix may be organized into supramolecular structures, which could impose selectivity on protein-binding that is of higher spatial order than possible with individual chains.

To test these ideas, we have used five FGFs (FGF1, 2, 6, 10 and 20) with distinct HS-binding sites and binding selectivity for structures in the polysaccharide [29,30]. These FGFs were expressed as N-terminal HaloTag fusions (Halo-FGFs) [31], which permitted specific fluorescent labelling. Measurement of the binding and diffusion of the Halo-FGFs to glycosaminoglycans in the pericellular matrix of fibroblasts revealed that there were very substantial differences between these FGFs in their level of binding, their spatial distribution and their diffusion. These data indicate that HS chains in pericellular matrix are organized over length scales far greater than that of a single chain, and that this serves to present distinct numbers and spatial patterns of binding sites for effectors, which in turn modulates the diffusion of the proteins.

## 2. Material and methods

### 2.1. Protein production

The FGFs and Halo-FGFs were produced exactly as described in detail previously [29,31]. HaloTag protein was produced by digestion of Halo-FGF20 with TEV protease and purified by anion-exchange on DEAE Sepharose Fast Flow (GE Healthcare, Buckinghamshire, UK). Protein concentrations were determined by measuring their absorbance at 280 nm using a NanoDrop 1000 spectrophotometer (Thermo Scientific, Leicestershire, UK).

### 2.2. Protein labelling

HaloTag and Halo-FGFs (0.5 µM) were incubated with 2.5 µM HaloTag TMR ligand (Promega UK Ltd, Hampshire, UK) in 100 µl phosphate-buffered saline (PBS: 2.7 mM KCl, 10 mM Na_2_HPO_4_, 1.8 mM KH_2_PO_4_ and 0.15 M NaCl, pH 7.4) at room temperature for 30 min, then kept on ice before use the same day. To determine the extent of labelling, TMR-dye-labelled Halo-FGFs were loaded onto a mini heparin agarose (BioRad, Hertfordshire, UK) column (20 µl) and washed with PBS containing 0.05% (v/v) Tween-20. The bound TMR-labelled Halo-FGFs were eluted with 2 M NaCl buffered with phosphate (PB: 2.7 mM KCl, 10 mM Na_2_HPO_4_, 1.8 mM KH_2_PO_4_, pH 7.4). The quantum yields were measured in a fluorescence spectrophotometer (Varian, Walton-on-Thames, UK) by excitation at 561 nm and emission from 565 to 700 nm.

### 2.3. Cell culture

Rat mammary (Rama) 27 fibroblasts were cultured with Dulbecco's modified Eagle's medium (Life Technologies, Paisley, UK) supplemented with 10% (v/v) fetal calf serum (Labtech International Ltd, East Sussex, UK), 4 mM l-glutamine (Life Technologies), 0.75% sodium bicarbonate (Life Technologies), 50 ng ml^−1^ insulin (Sigma-Aldrich, Dorset, UK) and 50 ng ml^−1^ hydrocortisone (Sigma-Aldrich), as described previously [32].

### 2.4. Cell labelling

Rama 27 cells were cultured on glass bottomed imaging dishes (CELLview Culture dish: 35 mm non-treated glass bottom, Greiner Bio-one, Stonehouse, UK) and fixed with 4% (w/v) paraformaldehyde dissolved in PBS. The fixed cells were washed with PBS three times and then incubated with 2 ml PBS containing 10 mg ml^−1^ BSA to block any remaining partially active fixative. The blocking medium was discarded after 15 min, and the fixed cells were incubated with 1.5 ml 10 nM TMR dye, 2 nM TMR-labelled HaloTag or 2 nM TMR-labelled Halo-FGFs for 30 min at 37°C. The excess TMR dye and TMR dye-labelled Halo-FGFs (TMR-Halo-FGFs) were removed by three washes with PBS. In competition experiments, the competitor was added along with the labelled Halo-FGF at concentrations indicated in the figure legends. Degradation of HS was achieved in fixed Rama 27 cells by incubation with 1 ml heparinase I, II and III (50 mU ml^−1^ each in 100 mM sodium acetate and 0.1 mM calcium acetate, pH 7.0; gift from Prof. Jerry Turnbull, University of Liverpool). Chondroitin sulfate (including dermatan sulfate) was degraded by incubation with 1 ml chondroitinase ABC (Sigma-Aldrich; 400 mU ml^−1^ in PBS). In both cases, cells were incubated with the enzymes overnight at 37°C prior to incubation with Halo-FGFs.

### 2.5. Microscopy and imaging

A LSM780 confocal microscope with an environmental control chamber (Zeiss, Jena, Germany) was used to acquire cell imaging data with a DPSS 561 nm excitation laser. For all cell imaging, a 63X oil immersion lens (Plan-Apochromat 63× 1.4 oil DIC M27) and a 15.03 Airy Units pinhole were used. Cell images (67.3 µm × 67.3 µm, 512 × 512 pixels, 16 bits) containing bright field and the red fluorescence channels were collected for the binding assays. Images were collected using identical microscope settings.

### 2.6. Fluorescence recovery after photobleaching

The fixed cells labelled with TMR-Halo-FGF1 (2 nM and 1 nM), TMR-Halo-FGF2 (2 nM), TMR-Halo-FGF6 (2 nM) and TMR-Halo-FGF10 (2 nM) were used for the FRAP experiments. The measurements were performed at 37°C. A square area (22.49 × 22.49 µm, 256 × 256 pixels, eight bits) was imaged six times with the 63X oil immersion lens, and then the selected 2.5 µm (radius) disc area was bleached with the 561 nm laser at full power for eight iterations (0.64 s in total). After that, another 195.6 s of images (994 images) were acquired to measure the fluorescence recovery. An area free of cells and a non-bleached area on the same cell were selected to determine the background (subtracted in quantifications) and correct the photobleaching caused by the excitation laser during imaging, respectively. The fluorescence intensities of these three selected areas from 0 to 197.2 s were extracted using ZEN 2012 software (Zeiss) for further analysis.

### 2.7. Data analysis

*Fluorescence intensity of the labelled cells:* the cell edges were automatically identified by using published Matlab codes [33], and the fluorescence intensities were averaged for each cell. The cell edges of low fluorescence-labelled cells were detected in the bright field channel image (electronic supplementary material, figures) and high fluorescence-labelled cells were detected in the fluorescence channel image. The Matlab program for cell edge detection can be downloaded from GitHub (https://github.com/hscsun/DrawCellEdges.git).

*FRAP data analysis:* the background fluorescence intensity (*I*_b_) was subtracted from both the bleached area (*I*) and non-bleached reference area (*I*_r_). The photobleaching was corrected by the reference area and *I*_c_ is the corrected fluorescence intensity of the bleaching area.2.1
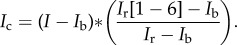
Note: *I*_r_[1–6] means the averaged fluorescence intensity of the reference area of the first six images; the other fluorescence intensities (*I*, *I*_b_, *I*_c_, *I*_cn_ and *I*_dcn_) are applied to any image in the frame, but they correspond to the same image number in both sides of the equation for each calculation (from frame 1 to 1000 in this FRAP experiment).

The fluorescence intensity of the bleaching area was normalized to the averaged fluorescence intensity of the first six images, where *I*_cn_ is the corrected and normalized intensity of the image.2.2
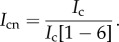
Note: *I*_c_[1–6] means the average of the first six corrected fluorescence intensities from equation (2.1).

To compare fluorescence recovery curves, the corrected and normalized fluorescence intensity of the first bleached image, *I*_cn_[7], was subtracted from the corrected fluorescence intensity of bleached area and the FRAP curve was normalized again, as in equation (2.2).2.3
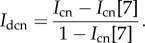
Note: here [7] means the seventh image (or the first image after bleaching) for the normalized and corrected fluorescence intensity.

The final recovery level (*I*_f_), the fluorescence intensity for the last measurement and half recovery time (*τ*_1/2_) were extracted from the corrected and normalized curve acquired from equation (2.3) by2.4
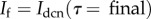
and2.5
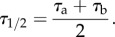
Note: *τ* means time; final is the time at which the last image was acquired in the actual experiments. *τ*_a_ is the time corresponding to the maximum value of the fluorescence intensities smaller than half of *I*_f_; *τ*_b_is the time corresponding to the minimum value of the fluorescence intensities larger than half of *I*_f_.

The radial profiles of the bleaching area were extracted using a published Matlab code [34], and the photobleaching was corrected for each analysed image as described in equation (2.1). The Matlab program for FRAP data analysis can be downloaded from GitHub (https://github.com/hscsun/ImagingDataAnalyzerForFRAP.git).

All calculations and image montages were done with Matlab R2014a.

Boxplots of the half recovery time and final recovery level of different Halo-FGFs were prepared in OriginPro v. 9. The data plot with standard deviation area was prepared using a published Matlab code [35].

## 3. Results and discussion

### 3.1. Labelling Halo-FGFs with TMR-Halo ligand dye

**Figure 1.**
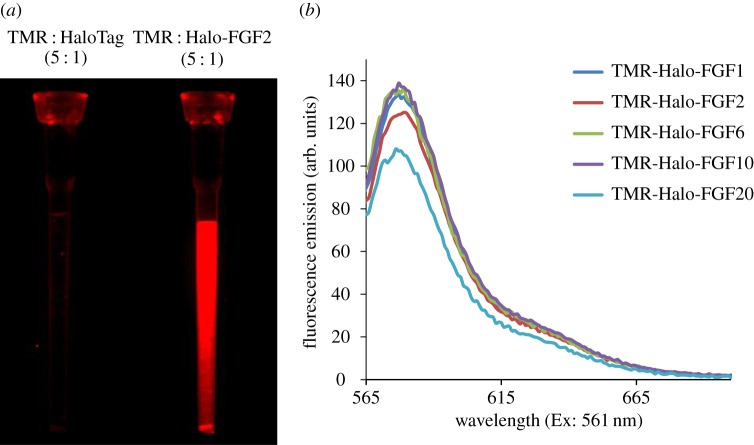
Conjugation and quantification of TMR dye-labelled Halo-FGFs. Halo-TMR dye was used to label HaloTag and Halo-FGFs at a ratio of 5 : 1 (mole/mole). The labelled HaloTag and Halo-FGFs were loaded onto a mini heparin column, which was subsequently washed with PBST. (*a*) The HaloTag and Halo-FGF2-loaded heparin columns were visualized under a red fluorescence filter (ImageQuant LAS 4000 imager, GE Healthcare). (*b*) The five TMR-labelled Halo-FGFs were loaded onto mini heparin-affinity chromatography columns, washed with PBS buffer and eluted with 2 M NaCl in the same buffer. The fluorescence intensities of the five purified Halo-FGFs were quantified in a fluorimeter by measuring the emission from 565 to 700 nm excited with 561 nm.

The N-terminal HaloTag fusion does not affect the binding of FGFs to heparin or their biological activity and they are efficiently expressed [31]. So, they provide a convenient means to prepare genetically encoded fluorescently labelled FGFs, whose excitation and emission properties can be altered by changing the HaloTag ligand [36]. We first tested whether the HaloTag TMR ligand dye interacted with heparin or grossly affected the interaction of the FGFs with heparin. A mixture of HaloTag and a fivefold excess of Halo-TMR dye was incubated for 30 min and loaded onto a mini heparin column. After three 50 µl washes with PBS containing 0.05% (v/v) Tween-20 (PBST) to remove the unbound dye, there was no red fluorescence detectable on the heparin column (figure 1*a*). This indicated that neither HaloTag nor the TMR-Halo ligand dye bound to heparin. In contrast, the heparin column loaded with TMR-labelled Halo-FGF2 gave strong red fluorescence (figure 1*a*), which demonstrated that Halo-FGF2 was labelled with TMR-Halo ligand dye and retained its heparin-binding properties. Following purification of fluorescent-dye-labelled Halo-FGFs on mini heparin columns, the bound TMR-Halo-FGFs were eluted with 2 M NaCl. The fluorescence emission curves of the purified Halo-FGFs demonstrated that the emission peak of TMR dye remained at 580 nm and that the emission curves of these Halo-FGFs were quite similar (figure 1*b*). Although there was a small difference of the fluorescence intensity for each TMR-Halo ligand labelled Halo-FGF, especially for Halo-FGF20, the results indicate that the labelling of different Halo-FGFs was very consistent and efficient.

### 3.2. Binding of different Halo-FGFs to Rama 27 fibroblast pericellular matrix heparan sulfate

Rama 27 fibroblasts were fixed with paraformaldehyde without permeabilization prior to imaging, so only extracellular binding sites will be measured [27]. This will also stop cellular biochemical processes, so binding of FGFs to pericellular matrix will not be affected by internalization.

#### 3.2.1. Halo-FGF2

**Figure 2.**
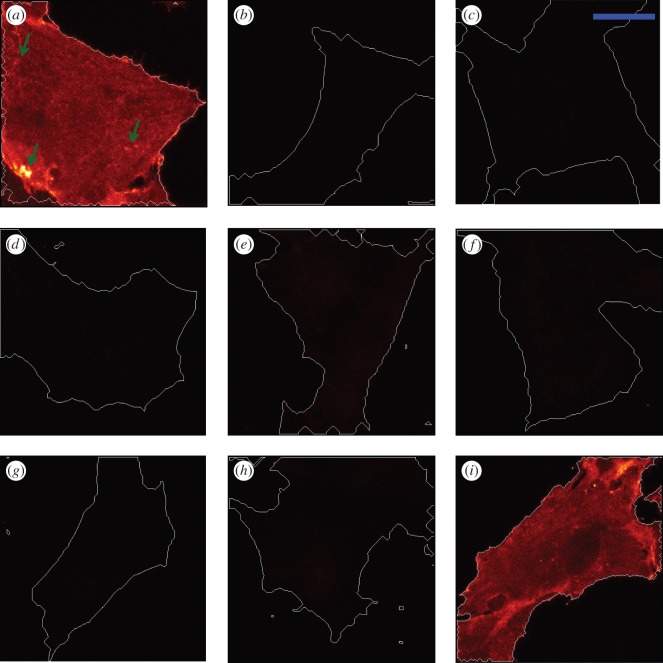
Halo-FGF2 binding to Rama 27 fibroblasts. The binding specificity of Halo-FGF2 to Rama 27 cells was determined by competing with unlabelled FGFs and heparin, and by digestion with heparinases and chondroitinase ABC. TMR-Halo-FGF2 (2 nM), Halo-TMR dye or TMR-HaloTag were used to label fixed Rama 27 fibroblasts for 30 min. The excess Halo-FGF2, Halo-TMR dye or TMR-HaloTag was removed by washing with PBS three times. The cell edges are highlighted with white lines. (*a*) TMR-Halo-FGF2 (2 nM). (*b*) Unlabelled cells imaged to show the autofluorescence. (*c*) Halo-TMR dyes (2 nM) to measure the non-specific binding of ligand dye to cells or glass dish. (*d*) TMR-HaloTag (2 nM) to determine the level of binding of HaloTag. (*e,f*) Cells incubated with 2 nM TMR-Halo-FGF2 and (*e*) 8 µM unlabelled Halo-FGF2 or (*f*) 8 µM FGF2. (*g*) 2 nM TMR-Halo-FGF2 and 4 µg ml^−1^ heparin. (*h*) Cells were incubated with heparinases I, II and III to remove heparan sulfate and then incubated with 2 nM TMR-Halo-FGF2. (*i*) Cells were incubated with chondroitinase ABC to digest chondroitin sulfate and then incubated with 2 nM TMR-Halo-FGF2. The corresponding bright field images are presented in electronic supplementary material, figure S1. Size of the scale bar is 20 µm.

The fixed Rama 27 fibroblasts were incubated with 2 nM Halo-FGFs to determine if their binding capacities to HS in the pericellular matrix of these cells differed. Halo-FGF2 strongly bound to Rama 27 fibroblasts (figure 2*a*). The bright spots show the heterogeneities in the distribution of the Halo-FGF2 (figure 2*a*, arrows).

To detect the autofluorescence from the imaged cells and the interactions of HaloTag with this pericellular matrix, a number of controls were used. The BSA-blocked cell dish was visualized by confocal microscopy. The cell edges were detected by the bright field image (electronic supplementary material, figure S1*b*), and no autofluorescence from the cells was observed in the fluorescence channel image (figure 2*b*). Using the same microscope settings, when TMR-Halo ligand alone and TMR-Halo ligand-labelled HaloTag were incubated with the fixed Rama 27 cells, the fluorescence was the same as observed with a BSA-blocked culture dish with cells; no red fluorescence was detectable (figure 2*c*,*d*). When the fixed cells were incubated with 2 nM TMR-labelled Halo-FGF2 and with either of two unlabelled competitors, 8 µM unlabelled FGF2 or Halo-FGF2, the binding was reduced to undetectable levels (figure 2*e*,*f*). These data indicate that non-specific binding of TMR-Halo ligand and of TMR-Halo ligand-labelled HaloTag protein was within the levels of background fluorescence and that the fluorescence observed with labelled Halo-FGF2 in Rama 27 pericellular matrix (figure 2*a*) was entirely owing to the FGF2 moiety of the Halo-FGF2.

To determine what Halo-FGF2 was binding to in the pericellular matrix of Rama 27 fibroblasts, a series of competition and enzyme digestion experiments were performed, again using the same microscope settings. Competition with heparin (4 µg ml^−1^ added with Halo-FGF2) abolished binding, and fluorescence was reduced to background levels (figure 2*g*). This indicates that FGF2 is probably bound to glycosaminoglycans of the pericellular matrix. Moreover, while heparin will effectively compete for binding of FGF2 to glycosaminoglycans, it still enables FGF2 to bind to the FGFR on these cells [37]. Therefore, Rama 27 fibroblasts were subjected to heparinase and chondroitinase ABC digestion to ascertain its binding partner(s). Incubation of fixed Rama 27 fibroblasts with heparinases I, II and III prior to the addition of Halo-FGF2 reduced the level of fluorescence to background levels (figure 2*h*). In contrast, chondroitinase ABC digestion of the cells did not appreciably alter the binding of TMR-Halo-FGF2 to Rama 27 cell (figure 2*i*). These data demonstrate that TMR-Halo-FGF2 is primarily bound to HS in the pericellular matrix of Rama 27 fibroblasts. Moreover, these results are consistent with previous data, which indicate that more than 99% of binding sites for FGF2 on Rama 27 fibroblasts are HS, and the FGFR less than 1% [27,38].

#### 3.2.2. Halo-FGF1

**Figure 3.**
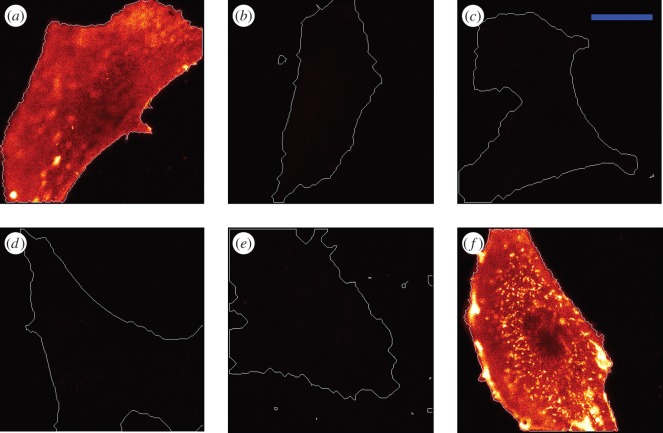
Halo-FGF1 binding to Rama 27 fibroblasts. The binding specificity of Halo-FGF1 to fixed Rama 27 fibroblasts was tested by competing with unlabelled FGF1 and heparin and by digestion with heparinase and chondroitinase ABC. (*a*) Cells were incubated with 2 nM TMR-Halo-FGF1 at 37°C for 30 min. (*b,c*) 2 nM TMR-Halo-FGF1 was added with (*b*) 8 µM unlabelled Halo-FGF1 or (*c*) 8 µM unlabelled FGF1. (*d*) TMR-Halo-FGF1 (2 nM) in the presence of 4 µg ml^−1^ heparin. (*e,f*) TMR-Halo-FGF1 binding to Rama 27 fibroblasts previously subjected to digestion with heparinase I, II and III, and chondroitinase ABC, respectively. The corresponding bright field images are presented in electronic supplementary material, figure S2. Size of the scale bar is 20 µm.

The binding of TMR-labelled Halo-FGF1 to Rama 27 cells was somewhat stronger than that observed for Halo-FGF2 (figures 2*a* and 3*a*). Because the labelling efficiencies of the Halo-FGFs are similar, this indicates that FGF1 at this concentration possesses more binding sites on these cells than FGF2. As for Halo-FGF2, the distribution of the fluorescence was not homogeneous (figure 3*a*). The lower fluorescence intensity in the centre of the cell was the result of the high focal plane of the plasma membrane in this region owing to the underlying cell nucleus. The same competition and enzyme digestion experiments performed with Halo-FGF2 were done with Halo-FGF1, to identify its binding partner(s) in Rama 27 pericellular matrix. Both unlabelled 8 µM Halo-FGF1 and FGF1 effectively competed with 2 nM TMR-Halo-FGF1 (figure 3*b*,*c*). Addition of 4 µg ml^−1^ heparin with TMR-Halo-FGF1 also abolished detectable binding of the latter to Rama 27 fibroblasts (figure 3*d*). Treatment of fixed Rama 27 cells with heparinases was similarly effective in reducing the binding of TMR-Halo-FGF1 below the limit of detection (figure 3*e*). However, digestion with chondroitinase ABC increased the level of fluorescence (figure 3*f*). The increase in binding of Halo-FGF1 observed after chondroitinase ABC treatment may indicate that removal of chondroitin sulfate changed the structure of ECM and somehow increased the number of available HS binding sites for FGF1 (figure 3*f*). Collectively, these data demonstrate that the detectable fluorescent Halo-FGF1, like the Halo-FGF2, is bound to the HS of the pericellular matrix of Rama 27 fibroblasts. Although FGF1 binds HS preferentially, it also binds dermatan sulfate more weakly [29], but dermatan sulfate binding sites are either not available or too weak in Rama 27 pericellular matrix, because chondroitinase ABC treatment increased, rather than decreased binding. Interactions with the FGFR are below the level of detection, which is consistent with the relative numbers of binding sites corresponding to HS and the FGFR established previously for FGF2 in these cells [27,38].

#### 3.2.3. Halo-FGF6

**Figure 4.**
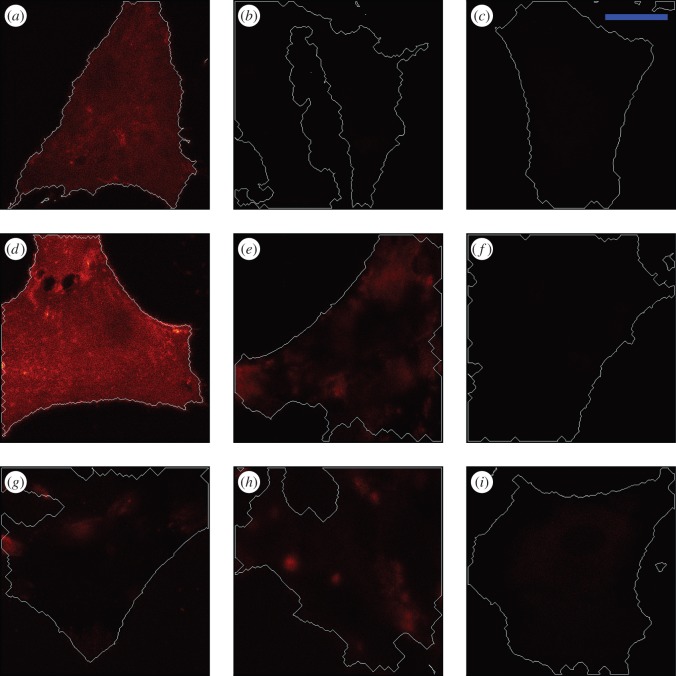
Binding specificity of Halo-FGF6 and Halo-FGF10 to Rama 27 fibroblasts. Halo-FGF6 and Halo-FGF10 were used to label fixed Rama 27 fibroblasts and heparin, and the enzyme digested fibroblast pericellular matrix was used to determine their binding specificity. (*a*) Rama 27 fibroblasts were incubated with 2 nM TMR-Halo-FGF6. (*b*) 2 nM TMR-Halo-FGF6 in the presence of 4 µg ml^−1^ heparin. (*c,d*) 2 nM TMR-Halo-FGF6 binding to Rama 27 fibroblast pericellular matrix digested with heparinase I, II and III, and chondroitinase ABC, respectively. (*e*) Rama 27 fibroblasts were incubated with 2 nM TMR-Halo-FGF10. (*f*) TMR-Halo-FGF10 (2 nM) in the presence of 4 µg ml^−1^ heparin. (*g,h*) TMR-Halo-FGF10 (2 nM) binding to Rama 27 fibroblasts pericellular matrix digested with heparinase I, II and III, and chondroitinase ABC, respectively. (*i*) 2 nM Halo-FGF10 binding to the pericellular matrix digested by both heparinase I, II and III, and chondroitinase ABC. The corresponding bright field images are presented in electronic supplementary material, figure S3. Size of the scale bar is 20 µm.

Halo-FGF6 bound only slightly less than FGF2 to fixed Rama 27 fibroblasts, and again the fluorescence was not homogeneous (figure 4*a*). No binding of Halo-FGF6 was observed on the fixed Rama 27 fibroblasts when TMR-Halo-FGF6 was added with 4 µg ml^−1^ heparin (figure 4*b*). Similar to FGF1 and FGF2, digestion of HS by heparinase decreased the binding of TMR-Halo-FGF6 to undetectable levels (figure 4*c*), whereas digestion of chondroitin sulfate and dermatan sulfate led to an increase in Halo-FGF6 binding to the cells, as seen with FGF1 (figure 4*d*). These results indicated that the detectable Halo-FGF6 was bound to HS in the pericellular matrix of Rama 27 fibroblasts. The number of these sites is similar to those recognized by FGF2, but chondroitin sulfate (or dermatan sulfate) would appear to prevent directly or indirectly some Halo-FGF6 binding to the HS in the pericellular matrix.

#### 3.2.4. Halo-FGF10

Halo-FGF10 only bound to some areas of the pericellular matrix, whereas in other areas, virtually no binding was detected (figure 4*e*). Thus, the binding of Halo-FGF10 to Rama 27 fibroblasts was characterized by very substantial heterogeneities. The binding sites on Rama 27 fibroblasts for FGF10 were also blocked by addition of 4 µg m^−1^ heparin, which effectively prevented FGF10 binding to fixed Rama 27 fibroblasts (figure 4*f*). Digestion of HS with heparinase I, II and III reduced the level of binding of Halo-FGF10 (figure 4*g*), but unlike Halo-FGF1, Halo-FGF2 and Halo-FGF6, did not abolish it (figures 2*h*, 3*e* and 4*c*). Moreover, digestion of chondroitin sulfate/dermatan sulfate with chondroitinase ABC also reduced the amount of bound Halo-FGF10 (figure 4*h*). These results indicated that Halo-FGF10 may bind to both HS and chondroitin sulfate in Rama 27 fibroblasts pericellular matrix. Therefore, a double digestion (heparinase and chondroitinase) was performed. When both sets of glycosaminoglycans were digested, the level of bound Halo-FGF10 was nearly undetectable (figure 4*i*), demonstrating that FGF10 does indeed bind to both chondroitin (dermatan) sulfate and HS.

### 3.3. Comparison of binding of Halo-FGFs to Rama 27 cell pericellular matrix heparan sulfate

**Figure 5.**
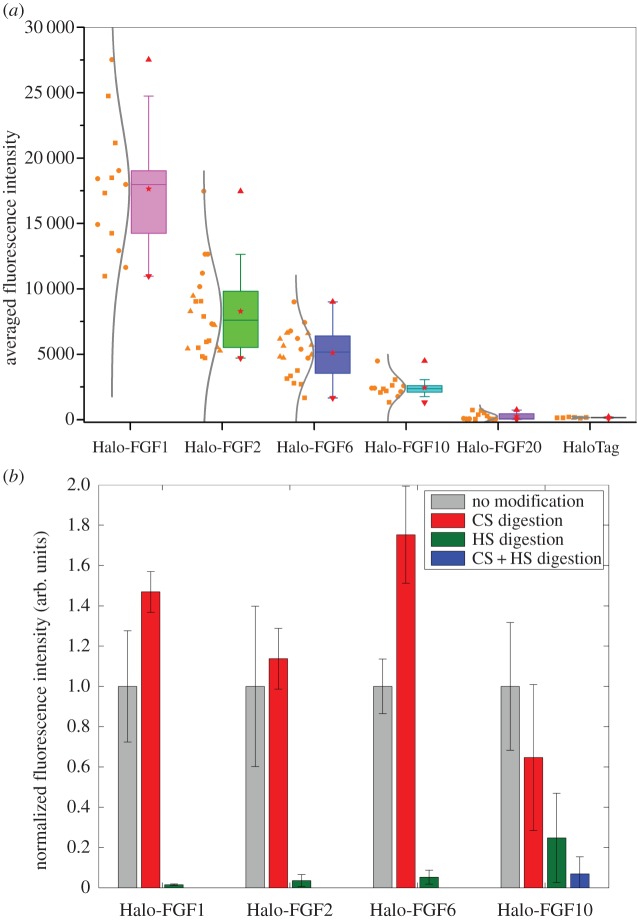
Quantification of binding of different Halo-FGFs to Rama 27 fibroblast pericellular matrix. (*a*) TMR-labelled Halo-FGF1, Halo-FGF2, Halo-FGF6, Halo-FGF10, Halo-FGF20 and HaloTag (all 2 nM) were incubated with fixed Rama 27 fibroblasts, as described in figures 2*a*, 3*a*, 4*a*,*e* and electronic supplementary material, figure S4*a,b*. The fluorescence in the highlighted cell area was averaged to quantify the level of binding of the FGF to Rama 27 pericellular matrix. Fluorescence intensities on different cells in the same set of dish and different sets of dishes were acquired and are shown as a box plot. Each symbol corresponds to independent dishes of cells measured on different days. (*b*) The binding intensities of Halo-FGF1, Halo-FGF2, Halo-FGF6 and Halo-FGF10 to Rama 27 fibroblasts pericellular matrix digested with heparinase I, II and III, and with chondroitinase ABC were quantified and normalized to the values obtained with untreated matrix.

Quantification of the level of binding of the Halo-FGFs to Rama 27 cell pericellular matrix revealed some marked differences. The level of binding was determined by calculating the averaged fluorescence intensity of the highlighted cell area to compare their binding capacities with the pericellular matrix. There were more binding sites for Halo-FGF1 than the other Halo-FGFs (figure 5*a*). Based on a Tukey *t*-test, the binding capacities of Rama 27 pericellular matrix for Halo-FGF2 and for Halo-FGF6 were also significantly different (*p* = 0.005, Tukey test) with Halo-FGF2 possessing more binding sites. In terms of binding intensity, Halo-FGF6 and Halo-FGF10 did not have significant difference (*p* = 0.08), but the distributions of Halo-FGF10 and the other three Halo-FGFs were clearly not the same (figure 5*a*). For example, Halo-FGF6 was more evenly distributed in pericellular matrix, with a level of heterogeneity similar to that seen with Halo-FGF2, whereas Halo-FGF10 only bound to specific areas of the pericellular matrix. In contrast, Halo-FGF20 bound extremely weakly, if at all, and it was consistently under the detection limit (figure 5*a*; electronic supplementary material, figures S4*a,b*).

These results differ from those obtained upon affinity chromatography of these FGFs to heparin [31]. The previous work indicated that Halo-FGF2, Halo-FGF1 and Halo-FGF10 could bind to heparin in 0.6 M NaCl, and 1 M or higher NaCl was required to efficiently elute them from heparin-affinity chromatography matrices. Both Halo-FGF6 and Halo-FGF20 could stably bind to heparin in 0.4 M NaCl. Though less Halo-FGF20 was bound than Halo-FGF6, this was due to a reduced capacity of the heparin affinity column for Halo-FGF20 [31]. This is consistent with the recent analysis of the structures in glycosaminoglycans recognized by FGFs, including FGF20, which requires long (more than 12 saccharides) sequences of sulfated saccharides for binding [30]. Such sequences would be rare in HS, though they may be present in HS of particular cells (e.g. syndecan-2 heparan suflate from liver) [39]. The present data also highlight that binding to heparin, which is far more sulfated than HS, does not reflect the binding capacity of HS, which is both less sulfated and more structurally diverse [1–3,40], which allows a far more selective interaction with individual proteins [7,41]. Moreover, the affinity of HS for a particular FGF may not predict the level of biding to the polysaccharide on the cell. Thus, the affinity of FGF1 for HS purified from Rama 27 cells is at least an order of magnitude lower than that of FGF2 [37], yet Halo-FGF1 binds to a greater extent than Halo-FGF2 (figure 5*a*).

The binding and competition data demonstrate that the detectable binding of the four Halo-FGFs is to glycosaminoglycans in the pericellular matrix. In the presence of heparin, these FGFs will interact with their receptor tyrosine kinase [42]. Thus, the absence of binding of Halo-FGF2 detected in the presence of heparin is in agreement with previous work, which showed that the number of HS-binding sites for FGF2 is several orders of magnitude greater than the number of receptors [27]. A similar difference is therefore likely to exist for FGF1, FGF6 and FGF10, because binding was not detected in the presence of heparin (figures 2*g*, 3*d* and 4*b,f*). Whereas Halo-FGF1, Halo-FGF2 and Halo-FGF6 interacted only with HS, Halo-FGF10 had a significant interaction with chondroitin sulfate (and/or dermatan sulfate) species on Rama 27 fibroblasts (figure 5*b*). FGF1 has previously been shown to interact with dermatan sulfate, but not chondroitin sulfate, whereas FGF7, which is in the same subfamily as FGF10, interacts weakly with both chondroitin sulfate and dermatan sulfate [29]. Consistent with the latter result, FGF10 has recently been shown to bind to chondroitin sulfate and dermatan sulfate [30]. In the case of Halo-FGF1, either the interaction with dermatan sulfate is too weak to be detectable or there is little dermatan sulfate with appropriate binding structures in Rama 27 cell pericellular matrix. In contrast, the interaction of Halo-FGF10 with chondroitin sulfate and/or dermatan sulfate on these cells is sufficiently strong to be detected (figure 5*b*).

The increase in binding observed with Halo-FGF1 and Halo-FGF6 upon chondroitinase ABC treatment of cells suggests that chondroitin sulfate may somehow mask HS binding sites for these Halo-FGFs. Whether such masking occurs directly or owing to bridging by endogenous proteins that bind both chondroitin sulfate and HS is not known. It is intriguing that the effect is not seen with Halo-FGF2, because this is in the same subfamily as FGF1, and the major difference in binding selectivity between these FGFs is that FGF1 readily binds tracts of sulfated saccharides containing 6-*O*-sulfated glucosamine with one of *N*-sulfated glucosamine or 2-*O*-sulfated iduronic acid, whereas FGF2 binds these poorly [5,7,43]. With respect to desulfated structures, the binding selectivity of FGF6 lies between that of FGF1 and FGF2, because FGF6 has a preference for structures containing 2-*O*-sulfated iduronate, but it does bind structures containing *N*-, and 6-*O*-sulfated glucosamine that lack sulfate on iduronate [30]. Thus, the masking effect of chondroitin sulfate on Halo-FGF1 and Halo-FGF6 binding to HS may be related to their interactions with such structures in HS.

The binding of all the Halo-FGFs was observed to be heterogeneous. This indicates that the distribution of binding sites for Halo-FGF1, Halo-FGF2 and Halo-FGF6 in HS and for Halo-FGF10 in HS and chondroitin sulfate are not evenly distributed across the pericellular matrix. This is consistent with similar imaging of gold nanoparticle-labelled FGF2 by photothermal heterodyne imaging (optical resolution) and by transmission electron microscopy [27,44]. Taken together, these data indicate that the previously observed clustering of FGF2 binding sites in HS of Rama 27 cell pericellular matrix may be a more general phenomenon, because it is seen here with four FGFs from three different subfamilies that possess different binding selectivity for HS [29,30]. This suggests that the binding sites for these FGFs are spatially organized in Rama 27 pericellular matrix, and this is likely to extend to supramolecular length scales (distance equivalent to several/many HS chains). Such organization would arise from the interaction of HS and (for FGF10) chondroitin sulfate/dermatan sulfate chains with their endogenous binding proteins, which for HS have been catalogued to at least 883 [4,45].

### 3.4. Detection of FGF diffusion by fluorescence recovery after photobleaching

The differences in the binding of Halo-FGF1, 2, 6, 10 and 20 to Rama 27 pericellular matrix, relate, at least in part, to differences in the structures these FGFs bind in HS (and chondroitin sulfate/dermatan sulfate for FGF10). It is established in some cases that the interaction of proteins with HS can control their movement in the extracellular space [15,27,46–48]. Therefore, to determine if the differences in HS binding may result in differences in movement in extracellular matrix, we measured the diffusion of Halo-FGF1, 2, 6 and 10 in Rama 27 pericellular matrix by FRAP.

The FRAP experiments employed the same labelling protocol as the imaging ones. Fixed cells were again used, because this allowed the measurement of the diffusion of each Halo-FGF in pericellular matrix to be made without any confounding effects that might have arisen owing to the movement of cells or of membrane. Paraformaldehyde reacts with primary amine groups and will not affect the binding structures of the FGFs used here, because these do not bind tracts of saccharides containing unsubstituted glucosamine [30] and in any event, such residues are rare in HS [49]. However, the fixative may cross-link endogenous multivalent HS-binding proteins and the core proteins of HS proteoglycans. This may then restrict movement of HS chains and diffusion in the membrane of the HS proteoglycan core proteins, both of which will restrict the freedom of the HS chains [27], though this effect may be less pronounced on glycosyl–phosphatidylinositol-anchored glypicans than transmembrane core proteins such as syndecans [50]. Another important feature of these experiments is that following the binding of Halo-FGFs to HS in the pericellular matrix, the cells were washed to remove unbound Halo-FGF. Trapping of FGF2 on HS in the extracellular matrix has been well documented [14,15,27,51–54] and, given a suitable density of HS-binding sites, is a general property of extracellular matrix [15,20,48,55]. As for FGF2 [27], Halo-FGF1, Halo-FGF2 and Halo-FGF6 bound to the pericellular matrix did not dissociate appreciably into the bulk culture medium over 270 s (electronic supplementary material, figure S5). Thus, because FRAP measurements were made in 197 s, dissociation into the bulk culture medium followed by re-association with HS in the pericellular matrix cannot contribute to the recovery of fluorescence. Instead, the recovery of fluorescence will be due to diffusion of these Halo-FGFs within the pericellular matrix.

**Figure 6.**
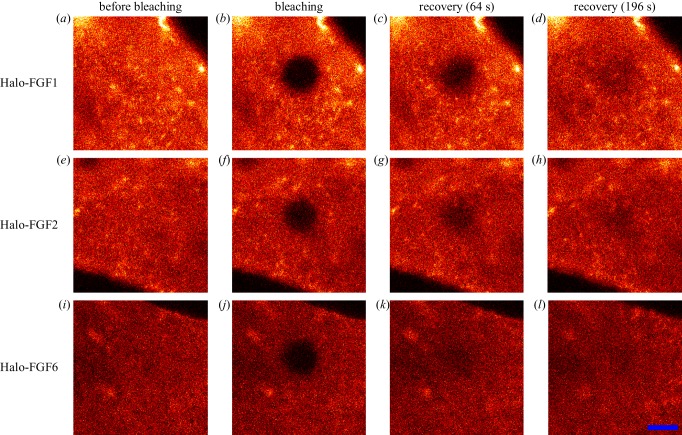
FRAP of Halo-FGF1, Halo-FGF2 and Halo-FGF6 in Rama 27 fibroblast pericellular matrix. Fixed Rama 27 fibroblasts were used to provide a pericellular matrix that could be probed with Halo-FGF1, Halo-FGF2 and Halo-FGF6. A 5 µm radius disc area on the cell was bleached by full power laser to measure the recovery of the fluorescence in the bleached area. (*a*,*e,i*) TMR-Halo-FGF1, -FGF2 and -FGF6 labelled cells before bleaching. (*b*,*f,j*) Same areas as (*a*,*e,i*), but following the bleaching of a 5 µm radius disc. (*c*,*g,k*) The partial recovery of fluorescence in the bleached area 64 s after bleaching. (*d*,*h,l*) Images acquired when the bleached area had recovered to a stable level (196 s). Size of the scale bar is 5 µm.

After the bleaching iterations, the selected area became dark (figure 6*a*,*b*,*e*,*f*,*i,j*). Recovery of fluorescence then occurred (figure 6*c*,*d*,*g*,*h*,*k*,*l*). These data demonstrate that the Halo-FGFs were able to diffuse between the bleached and surrounding areas of pericellular matrix in fixed Rama 27 cells. Thus, while these Halo-FGFs were clearly trapped within the pericellular matrix (electronic supplementary material, figure S5), they were able to diffuse within it. Movement of nanoparticle-labelled FGF2 has similarly been evidenced before by photothermal imaging, tracking and raster image correlation spectroscopy [27,44]. Earlier work also demonstrated that FGF2 trapped on HS in extracellular matrix was mobile [15]. Thus, the present data demonstrate that the movement of proteins bound to HS and trapped in extracellular matrix is likely to be a more general phenomenon.

### 3.5. Quantification of diffusion of Halo-FGF1, Halo-FGF2, Halo-FGF6 and Halo-FGF10

**Figure 7.**
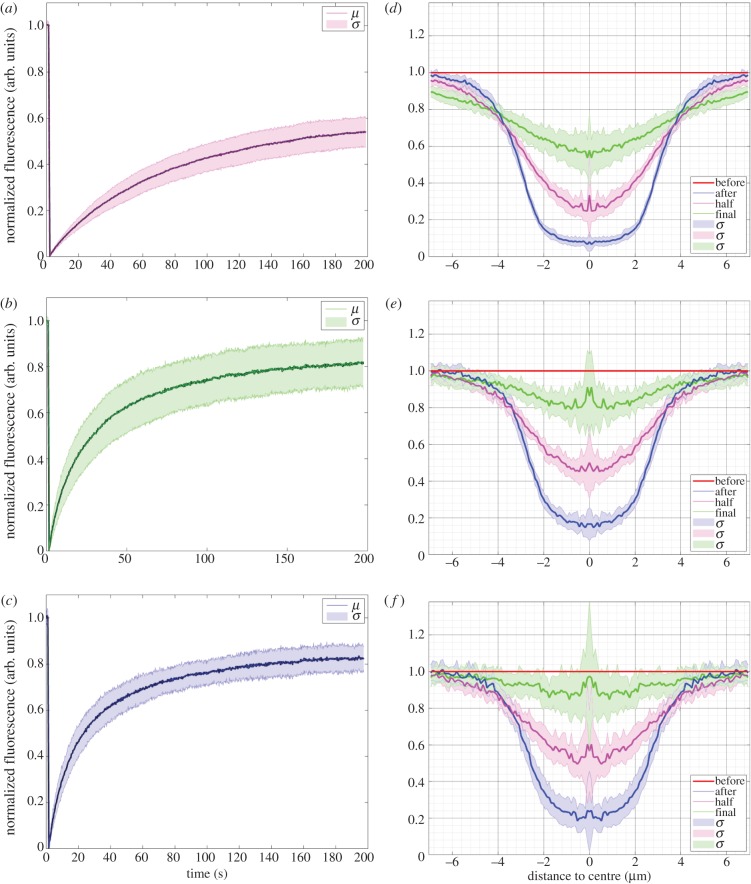
Fluorescence recovery curves and recovery radial profiles of Halo-FGF1, Halo-FGF2 and Halo-FGF6 in Rama 27 fibroblast pericellular matrix. The fluorescence intensity of the bleaching area was analysed, as described in materials and methods, to identify the different recovery patterns. The radial profile of the bleached area was extracted from the imaging data to reflect how the FGFs exchanged between the bleached area and the surrounding non-bleached pericellular matrix. (*a*,*b,c*) The normalized fluorescence intensities of (*a*) Halo-FGF1, (*b*) Halo-FGF2 and (*c*) Halo-FGF6 in the bleached area were plotted against time (average of 10 measurements for Halo-FGF1, 17 measurements for Halo-FGF2 and 28 measurements for Halo-FGF6). (*d*,*e,f*) The radial profiles of the bleached area before bleaching, immediately after bleaching, when fluorescence had reached half the final recovery value and at final recovery were extracted from the imaging data. Multiple repeats were applied to acquire the standard deviation. The mean of radial profiles for each FGF was plotted with standard deviation area against the distance to the centre of the bleached disc area (18 measurements for Halo-FGF1, 23 for Halo-FGF2 and 17 for Halo-FGF6). *μ* is the mean value of multiple fluorescence intensity curves for each FGF; *σ* is the standard difference; ‘before’ is before bleaching; ‘after’ is the image immediately after bleaching; ‘half’ is the time when the fluorescence was recovered to half of the final recovery level; ‘final’ is the time for the last measurement.

The fluorescence intensity of the bleached area during recovery was quantified as the normalized fluorescence (Materials and methods). In the case of Halo-FGF1, recovery was partial after 64 s and still not complete by 196 s (figure 6*c*,*d*; electronic supplementary material, videos S7 and S8). The fluorescence recovery curve shows that Halo-FGF1 fluorescence in the bleached area recovered relatively slowly and by 196 s only half the fluorescence was recovered (figure 7*a*). The decrease of fluorescence intensity of the reference area was due to the photobleaching by the imaging laser (electronic supplementary material, figure S6*a,b*), because Halo-FGF1, Halo-FGF2 and Halo-FGF6 could be trapped in the pericellular matrix for more than 4.5 min (electronic supplementary material, figure S5), as discussed above. The recovery of fluorescence was greater for Halo-FGF2, although the recovery was not complete after 196 s (figure 6*g*,*h*; electronic supplementary material, video S9). Quantification of the recovery of Halo-FGF2 fluorescence demonstrates that this is substantially faster than that of Halo-FGF1 and the final level of fluorescence, 80%, was higher (figure 7*b*). The fluorescence of Halo-FGF6 recovered similar to that of Halo-FGF2 (figure 6*k,l*; electronic supplementary material, video S10). The rate of fluorescence recovery of Halo-FGF6 was somewhat faster than Halo-FGF2, though the level of recovery attained after 196 s was similar (figure 7*c,e*). The weaker photobleaching for Halo-FGF2 might suggest that the bleached Halo-FGF2 and Halo-FGF6 during imaging could be quickly exchanged into the surrounding areas that were not imaged (electronic supplementary material, figure S6*c,d*).

**Figure 8.**
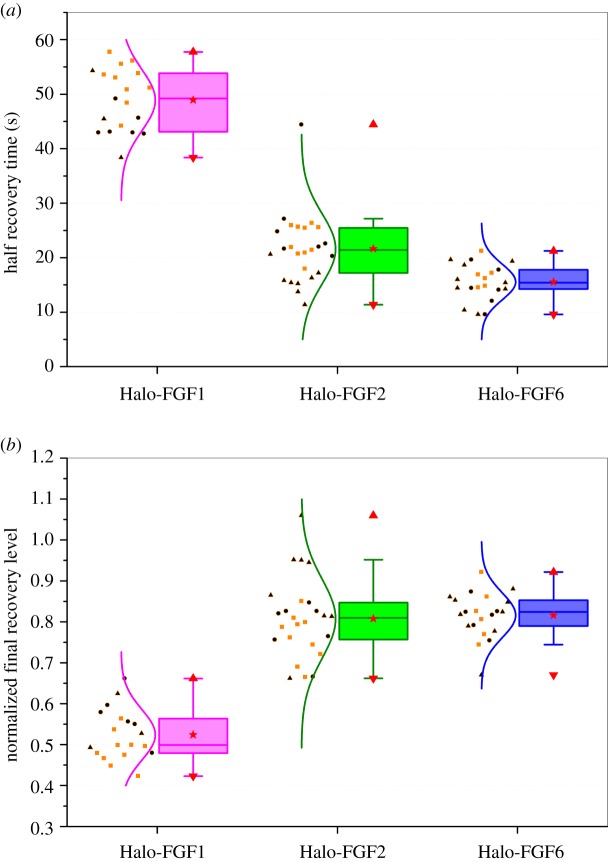
Quantification of moving speed and mobile/immobile fractions of Halo-FGF1, Halo-FGF2 and Halo-FGF6 on Rama 27 fibroblasts. The half recovery time and final recovery level were extracted from each fluorescence recovery curve, as described in materials and methods. (*a*) The half recovery times for Halo-FGF1, Halo-FGF2 and Halo-FGF6 were plotted to compare their diffusion speeds in the pericellular matrix. Each half recovery was extracted from one FRAP experiment, which shows the time it took to recover to half of the final fluorescence intensity in each fluorescence recovery curve. (*b*) Normalized final recovery levels of the three FGFs were used to determine the ratio of mobile and immobile FGF in the pericellular matrix. The final recovery level shows the mobile fraction, and the immobile fraction is its difference from 100%. Each symbol corresponds to independent dishes of cells measured on different days.

The fluorescence recovery curves (figure 7*a–c*) allowed the calculation of the half recovery time, which is directly related to the movement of molecules in the FRAP experiments and the relative proportions of mobile and immobile Halo-FGF. The half recovery times demonstrated that Halo-FGF1 diffused more slowly in the pericellular matrix of Rama 27 fibroblasts than Halo-FGF2 or Halo-FGF6 (figure 8*a*). Moreover, Halo-FGF6 had the shortest half recovery time (16 s), which was significantly (*p* = 0.0008, Tukey test) faster than that of Halo-FGF2 (22 s) and of Halo-FGF1 (49 s). Thus, the difference of final level of recovered fluorescence and the initial fluorescence is indicative of the fraction of immobile Halo-FGFs. Only 52% of Halo-FGF1 was mobile, whereas 81% of Halo-FGF2 and 82% of Halo-FGF6 were mobile (figure 8*b*). Previous work demonstrates that FGF2 that appears immobile at the resolution of a confocal microscope will in fact be undergoing confined motion, diameter approximately 100 nm [27]. Like Halo-FGF2, Halo-FGF1 and Halo-FGF6 are also bound to HS in the pericellular matrix. Although there are clear differences in the distribution and number of their available binding sites on HS chains, it seems reasonable to suggest that the immobile fraction of Halo-FGF1 and Halo-FGF6 are also undergoing similar confined motion.

Analysis of the movement of FGF2 at the single molecule level revealed that it undergoes different types of diffusive motion over different length scales. To see if some insight could be gained from the present average measurements of Halo-FGF diffusion into the types of movements the FGFs underwent, the fluorescence of the bleached area and surrounding unbleached area were determined as a series of radial profiles, diameter 14 µm. These analyses are presented as the radial profile at selected times: before bleaching, after bleaching, at the time corresponding to half recovery of the final fluorescence and at final recovery. The results show that the radial profile after bleaching (figure 7*d–f*, blue lines) is ‘U’ shaped, but, as the bleached area recovered, the profile (figure 7*d–f*, pink lines) it became more ‘V’ shaped. Moreover, for Halo-FGF1, as the recovery profile of the bleached area (2.5 µm radius) increased, there was a small decrease in fluorescence in the surrounding unbleached area (figure 7*d*, pink line and green line). Together, this suggests that the majority of the movement of the Halo-FGFs at these time scales is over 1 µm or less, corresponding to the confined and simple diffusive motion observed previously with FGF2, and that FGF1 may undergo comparatively less fast and directed diffusion [27]. In contrast, the half recovery profiles of Halo-FGF2 and Halo-FGF6 (figure 7*e*,*f*, pink lines) were more ‘U’ shaped, and the fluorescence of the surrounding unbleached areas was not much affected during recovery (figure 7*d*, pink line). Moreover, the final recovery profiles of Halo-FGF2 and Halo-FGF6 were close to that seen before bleaching (figure 7*e*,*f*, green lines). These data are consistent with the previous demonstration that FGF2 can undergo fast and directed diffusion in addition to confined and simple diffusive motion, and it would appear that Halo-FGF6 may undergo similar types of movement.

**Figure 9.**
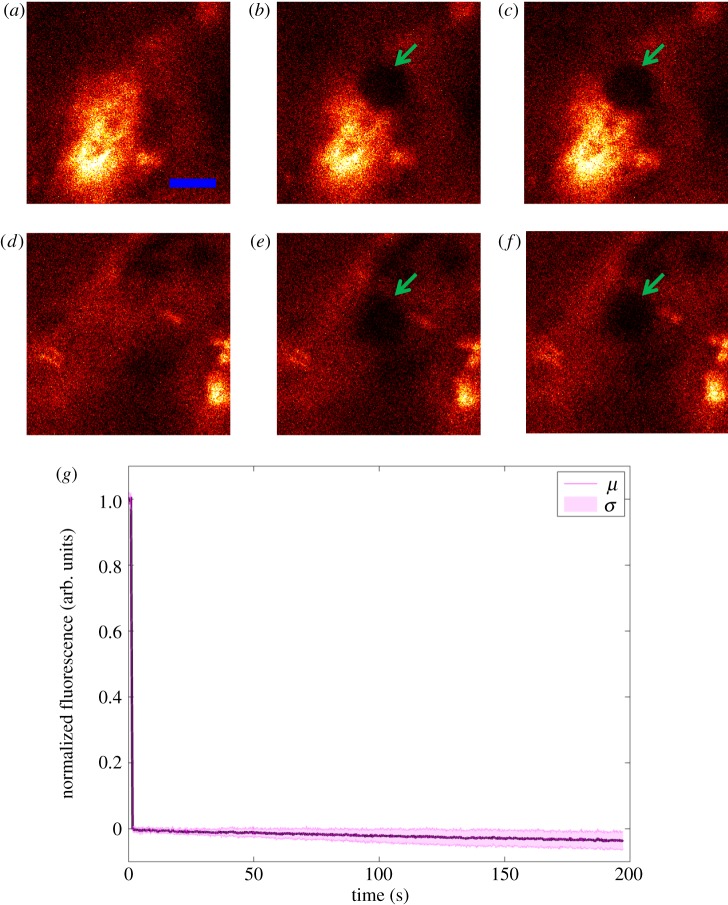
Diffusion of Halo-FGF10 in Rama 27 fibroblast pericellular matrix. Fixed Rama 27 fibroblasts were used to provide a pericellular matrix for Halo-FGF10 binding. A 5 µm radius disc area on the cell was bleached by full power laser to measure the recovery of the fluorescence in the bleached area. The fluorescence intensity of the bleached area was extracted to detect the diffusion of TMR-Halo-FGF10 in the pericellular matrix. (*a,d*) TMR-Halo-FGF10 labelled cells (two areas with different binding intensities) before bleaching. (*b,e*) Same areas as (*a,d*), but following the bleaching of a 5 µm radius disc. (*c,f*) The partial recovery of fluorescence in the bleached area 196 s after bleaching. (*g*) The normalized fluorescence intensities of Halo-FGF10 in the bleached area were plotted against time (average of 10 measurements). Size of the scale bar is 5 µm.

Because the distribution of Halo-FGF10 in Rama 27 fibroblast pericellular matrix was very heterogeneous, FRAP experiments were conducted to determine the diffusion of Halo-FGF10 in both areas of high (figure 9*a–c*) and lower binding (figure 9*d–f*). As for Halo-FGF1, Halo-FGF2 and alo-FGF6, a small area of the cells was bleached, and the fluorescence recovery was measured over the following 196 s (figure 9*a–c,d–f*). Compared with the image acquired immediately after bleaching, there was no obvious recovery of fluorescence after 196 s (figure 9*b,c*,*e,f*). The averaged fluorescence recovery curve demonstrates that the TMR-Halo-FGF10 in the bleaching area did not exchange appreciably with the TMR-Halo-FGF10 outside the bleached area (figure 9*g*). These data suggest that FGF10 does not dissociate readily from the HS, chondroitin sulfate and dermatan sulfate chains it is bound to. Interestingly, the thermal shift assay used to identify its selectivity for sulfation patterns with a library of modified heparins shows that rather than equilibrating between bound and unbound forms, FGF10 appears to partition into two populations, FGF10 and FGF10 bound to heparin [30]. This is consistent with a very slow dissociation of FGF10 from heparin, because faster dissociation would enable exchange of FGF10 molecules on the heparin, and so an averaging of the measured thermal stability of bound and unbound species. Work in two development models where FGF10 has a role in epithelial morphogenesis, in lung and salivary gland morphogenesis, also indicate that FGF10 bound to glycosaminoglycans does not readily dissociate and that FGF10 diffusion requires either suboptimal binding structures or the action of heparanase [21,56].

The substantial differences in diffusion observed between Halo-FGF1, Halo-FGF2 and Halo-FGF6 may be a consequence of differences in the number and spatial organization of their respective binding sites on HS chains. Alternatively, the much greater level of binding of Halo-FGF1 may reduce its mobility, owing to crowding and a consequent lower availability of free binding sites. To distinguish between these possibilities a lower concentration of Halo-FGF1 was used to measure its diffusion.

### 3.6. Effect of changing the concentration of Halo-FGF1

**Figure 10.**
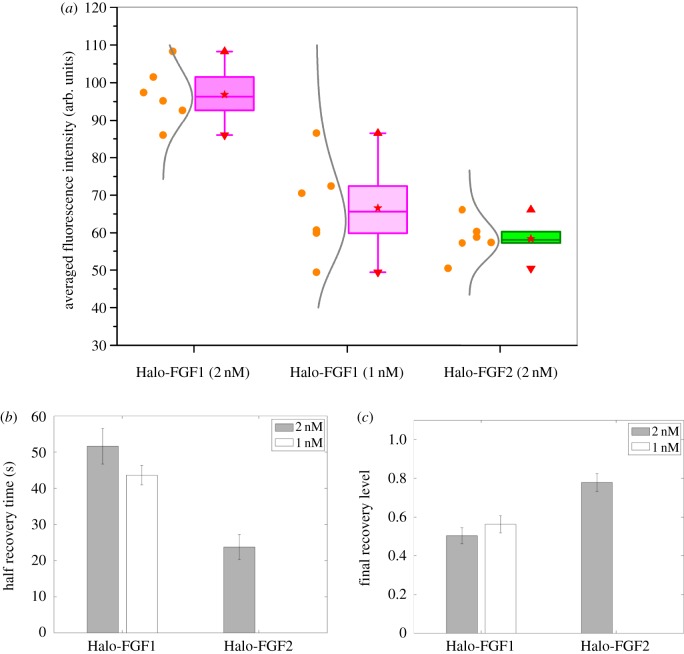
Effect of concentration of Halo-FGF1 and of Halo-FGF2 on their binding and diffusion in Rama 27 fibroblast pericellular matrix. TMR-Halo-FGF1 (1 nM) was used to label fixed Rama 27 fibroblasts to identify any dependence of their level of binding and their diffusion on concentration. The FRAP experiments with TMR-Halo-FGF1 (2 nM) and TMR-Halo-FGF2 (2 nM) were carried out at the same time and are shown in figures 7 and 8. Six FRAP experiments are included for each sample. (*a*) Binding intensities of Halo-FGF1 (1 and 2 nM) and Halo-FGF2 (2 nM) at the areas for FRAP measurements. (*b*) The moving speed (half recovery time) of different concentration of Halo-FGF1 and Halo-FGF2 in the matrix. (*c*) The mobile/immobile fraction (Final recovery level) of different concentration of Halo-FGF1 and Halo-FGF2 in fixed Rama 27 fibroblasts pericellular matrix.

The level of bound TMR-Halo-FGF1 was changed by halving the concentration of Halo-FGF1 added to fixed Rama 27 cells, which reduced the fluorescence intensity to levels similar to that observed with 2 nM TMR-Halo-FGF2 (figure 10*a*). However, at the lower level of binding of Halo-FGF1, the recovery of fluorescence following bleaching was similar to that observed with 2 nM Halo-FGF1. The half recovery time for 1 nM Halo-FGF1 was 45 s, and only 50% of the fluorescence was recovered. Consequently, reducing the amount of Halo-FGF1 bound to the HS in pericellular matrix by a factor of 2 had no strong effect on the diffusion speed of the Halo-FGF1 or on the relative proportions that were mobile and immobile (figure 10*b,c*). These results indicate that the slower diffusion observed with 2 nM Halo-FGF1 is unlikely to be due to the larger amount of Halo-FGF1 bound to HS in the pericellular matrix. Instead, the slower diffusion of Halo-FGF1 is more likely to be due to differences in the number and spatial organization of these binding sites, and the rate of association and disassociation of the FGF1 from them. Thus, the diffusion measurements suggest Halo-FGF1 is less mobile in pericellular matrix than FGF2 or FGF6 and it moves in smaller steps. If there was a focal source of FGFs, then FGF1 would form shorter and steeper gradients than FGF2 and FGF6 in Rama 27 fibroblast matrix.

### 3.7. Binding and movement of fibroblast growth factors in extracellular matrix

The expansion of the FGF family is associated with an increase in the complexity of multicellular organisms, highlighting its importance in mediating cell communication in development and homeostasis [57–59]. FGFs in a subfamily are more closely related in amino acid sequence and in function than FGFs in different subfamilies. The functional relations are evidenced, for example, by the selectivity of FGFs in different subfamilies for isoforms of the FGFR [42,60], for the patterns of sulfated sugars they bind in HS, and for the number and location of HS binding sites on the FGF [29,30,61]. Previous work with FGF2 demonstrated that its diffusion in pericellular matrix of Rama 27 fibroblasts was controlled by the spatial organization of its HS-binding sites [27]. This raises an important question: whether the diffusion of other HS-binding effectors, with different selectivity for patterns of sulfated sugars, also possess heterogeneous networks of binding sites that control their diffusion. To tackle this question, we have used five FGFs from four different subfamilies, with well-characterized HS-binding properties. This allows the effects of subtle differences between members of the same subfamily (FGF1 subfamily: FGF1 and FGF2) and more substantial differences between members of different subfamilies (FGF6 is in the FGF4 subfamily, FGF10 in the FGF7 subfamily and FGF20 in the FGF9 subfamily) to be measured.

There are differences between the diffusion of FGFs occurring in the experiments described here and *in vivo*. First, the Halo-FGF is bound to pericellular matrix and any unbound ligand is removed by washing. Thus, unlike *in vivo*, there is no source of diffusing ligand. Second, because the cells are fixed, receptor-mediated endocytosis cannot occur, so there is no sink to remove ligand. Therefore, the binding experiments (figures 2–4) provide a snapshot of the distribution of binding sites on glycosaminoglycans in pericellular matrix. The FRAP experiments measure the movement of the FGF owing to its dissociation and re-association to sites on glycosaminoglycans, without any effects of concentration gradients or cell biochemistry (membrane protein movement, membrane flow and cell movement).

There are a large number of binding sites for FGFs and other HS-binding proteins on the polysaccharide in pericellular matrix; for FGF2 in Rama 27 fibroblasts these amount to 3 × 10^6^ sites per cell [27]. The five FGFs used here preferentially bind different structures in HS and, perhaps unsurprisingly, their level of binding differed considerably; FGF1 bound to the greatest extent, whereas the binding of FGF20 was undetectable, because it was within the threshold of background fluorescence (figures 2–4; electronic supplementary material, figure S4). In all cases, the distribution of the FGFs was heterogeneous (figures 2–4), indicating that their binding sites are not evenly distributed in pericellular matrix. This has been shown previously for FGF2 over length scales ranging from 10 nm to several micrometres in the same cells [27,44]. The clustering of HS proteoglycans in lipid rafts would be one mechanism that could contribute to the heterogeneous distribution of HS-binding sites [62,63]. Other mechanisms may operate in parallel. For example, interactions of transmembrane proteoglycans (e.g*.* syndecans [64]) with the cytoskeleton through their cytoplasmic domains may lead to their localization to particular membrane microdomains.

The present data demonstrate that the heterogeneous distribution of binding sites observed previously with nanoparticle-labelled FGF2 [27,44] and in experiments with radiolabelled FGF2 [65] is likely to be a more general phenomenon, because it was observed here also with FGF1, FGF6 and FGF10. One interpretation is that the HS chains possessing binding sites for a particular protein (FGFs in the present case) are at least in part differently localized in pericellular matrix, through, for example, the various clustering above-discussed mechanisms. However, this interpretation is likely to be too simplistic. For HS, there are 883 extracellular proteins that bind it in the human proteome [4,45]. Thus, the subset of the HS-binding proteins expressed by Rama 27 fibroblasts will have a substantial portion of their binding sites engaged with HS. Consequently, the HS-binding sites available to a particular FGF (3 × 10^6^ for FGF2 [27]) are likely to be less than the total possible binding sites. Moreover, these HS-binding proteins also have very extensive networks of protein–protein interactions [4,45], which will influence their protein–polysaccharide interactions. One consequence of this multiplicity of interactions is that there are many free binding sites for exogenously added proteins on HS (figures 2–4) and there are many free binding sites on endogenous HS-binding proteins for exogenously added polysaccharide [66]. Thus, pericellular matrix is not at equilibrium and the ingress of an HS-binding protein may perturb a wide range of interactions. Such perturbations may involve substantial changes in the three-dimensional structure of HS chains. For example, a number of HS-binding proteins are multivalent, that is they have more than one binding site for the polysaccharide [28–30,61,67–70]. A recent biophysical analysis of brushes of HS chains demonstrated that some HS-binding cytokines and growth factors with multiple binding sites are able to cross-link the chains [28]. Because HS-binding matrix proteins such as collagens and fibronectin have multiple binding sites for the polysaccharide, it seems reasonable that they too will in some instances cross-link HS chains. Thus, the HS chains in pericellular matrix are likely to be engaged in large-scale supramolecular networks, which may ultimately be responsible for the heterogeneous distribution of binding sites and through which the Halo-FGFs diffuse.

The FRAP data for the four FGFs with detectable binding show that they move differently in Rama 27 fibroblast pericellular matrix (figures 7–10). In the case of FGF1 and FGF2, the slower movement of the former may be explained by its larger number of binding sites. Within the FGF1 subfamily, FGF1 binds to any disulfated saccharide structure of degree of polymerization (dp) 4 or longer, whereas FGF2 requires *N*-sulfate and 2-*O*-sulfated groups [5,7,43,71]. Thus, even taking into account occupation of some sites by endogenous proteins, the greater promiscuity of FGF1 is likely to explain why Halo-FGF1 binds Rama 27 pericellular matrix to a greater extent than Halo-FGF2. The larger number of sites in HS that FGF1 can bind may also underlie its more restricted mobility; a greater density of binding sites would reduce the distance the protein can travel in a given time, because the likelihood of rebinding will be greater. Indeed, binding site density and clustering have been shown to prevent effective dissociation of HS-binding proteins such as FGF2 from pericellular matrix and are likely to alter the distance a protein can travel within pericellular matrix before re-binding [15]. The differences in movement of the other FGFs would then similarly reflect their selectivity for binding structures in HS and how the available binding structures are presented. In the extreme, as seen with FGF10, the FGF does not diffuse appreciably over the time of the FRAP measurement. In such instances, the movement of the HS-binding protein would require additional mechanisms. This could be provided by heparanase, an extracellular *β* glucuronidase, which cleaves HS chains in their transition domains. This would release cargoes of S-domains and bound protein, as shown for FGF2 in a skin wound healing model [72]. Indeed, heparanase has been shown to be important for the stimulation of ductal morphogenesis by FGF10 in salivary gland [56].

## 4. Conclusion

The selectivity of FGFs for different binding structures in glycosaminoglycans provides a means to probe the distribution of these binding sites in Rama 27 cell pericellular matrix and to determine the effect this has on the diffusion of the FGFs. The results show that protein-binding sites in HS (and chondroitin sulfate/dermatan sulfate for FGF10) of pericellular matrix are not homogeneously distributed. A number of different mechanisms are likely to regulate the distribution of these binding sites, including the biosynthesis of the HS chains, the localization of core proteins in membrane microdomains and the interactions of the polysaccharide chains with endogenous HS-binding proteins. The high multiplicity of interactions, both between proteins and polysaccharide and between the polysaccharide-binding proteins themselves [4] (reviewed in [1,2]), is likely to produce a dynamic network of interlinked molecules. This would then be responsible for the long-range (supramolecular) structure of the pericellular matrix, which determines its spatial binding capabilities for individual proteins. Such a structure would be sensitive to perturbations, such as the ingress of an HS-binding protein from a neighbouring cell (in the same or different tissue compartment), and can control the diffusion of such effector proteins. Supramolecular structure in extracellular matrix has been shown in cartilage [73], where there are also definitive structural and functional differences between the pericellular matrix of chondrocytes, and the territorial and inter-territorial matrices that are more distant from the cells. Thus, although extracellular matrix in cartilage is specialized, in other tissues, an analogous situation may exist, where pericellular, extracellular and basement membrane matrices may exhibit different types of supramolecular structure and consequently have different functions.

## Supplementary Material

Sun et al FGF diffusion ECM SI.pdf

## Data accessibility

Software written for image analysis is available as follows:

The program for ell edge detection can be downloaded from GitHub at https://github.com/hscsun/DrawCellEdges.git.

The program for FRAP data analysis can be downloaded from GitHub at https://github.com/hscsun/ImagingDataAnalyzerForFRAP.git.

The raw imaging data used for data analysis are stored in OMERO, as described in the electronic supplementary material, table S1. The original image files are accessible via the Centre for Cell Imaging's Omero database, using the URL provided in the electronic supplementary material.
