# Supplementary material for: Selectivity in glycosaminoglycan binding dictates the distribution and diffusion of fibroblast growth factors in the pericellular matrix
Source: Open Biol. 2016 Mar 23;6(3):150277. doi: 10.1098/rsob.150277 (PMC4821244; doi:10.1098/rsob.150277)
Supplement: Sun et al FGF diffusion ECM SI.pdf [file rsob150277supp1.pdf]

Selectivity in glycosaminoglycan-binding dictates the distribution and diffusion of fibroblast growth factors in the pericellular matrix

Changye Sun[1], Marco Marcello[2], Yong Li[1], David Mason[2], Raphaël Lévy[1] and David G. Fernig[1]

[1]Department of Biochemistry, Institute of Integrative Biology, University of Liverpool, Liverpool, UK L69 7ZB

and

[2]Centre for Cell Imaging, Institute of Integrative Biology, University of Liverpool, Liverpool, UK L69 7ZB

Corresponding Author:

David G. Fernig, Department of Biochemistry, Biosciences Building, University of Liverpool, Crown Street, Liverpool L69 7ZB, UK. Tel.: +441517954471; Fax: +441517954406; E-mail: dgfernig@liverpool.ac.uk.

Supplementary Electronic Information

Contents **Page**

Figure S1: The brightfield images corresponding to the images in Fig. 2. 3

Figure S2: The brightfield images corresponding to the images in Fig. 3. 4

Figure S3: The brightfield images corresponding to the images in Fig. 4. 5

Figure S4: Binding of Halo-FGF20 and HaloTag to Rama 27 fibroblasts. 6

Figure S5: Measurement of dissociation of Halo-FGF1, Halo-FGF2 and Halo-FGF6 into the bulk culture medium. 7

Figure S6: Photobleaching of TMR-Halo-FGFs during FRAP experiments. 8

Figure S7: FRAP movie for 2 nM Halo-FGF1. 8

Figure S8: FRAP movie for 1 nM Halo-FGF1. 8

Figure S9: FRAP movie for 2 nM Halo-FGF2. 8

Figure S10: FRAP movie for 2 nM Halo-FGF6. 8

Table S1: Raw imaging data list in OMERO. 9

Introduction

The bright channel images in Figs S1, S2, S3 and S4 were acquired to show the locations and profiles of the cell. The edges of the cells with low fluorescence intensity were highlighted as described in the methods. Fluorescence and brightfield channels of Halo-FGF20 (Figs. S4 *a* and *c*) and HaloTag (Figs. S4 *b* and *d*) show the binding of Halo-FGF20 to the Rama 27 fibroblast pericellular matrix. Since Halo-FGF20 exhibited weak non-specific binding to the culture dish (Fig. S4*a*), any binding of Halo-FGF20 was within this background signal.

The fluorescence intensities in a same area were quantified for Halo-FGF1, Halo-FGF2 and Halo-FGF6 over 4.5 min (Fig. S5). No change of the fluorescence intensities for these three Halo-FGFs was detected, indicating that the FGFs are effectively trapped in pericellular matrix over this time and do not dissociated appreciably into the bulk culture medium. The fluorescence intensities of reference areas during the FRAP experiments were also quantified. The FGF trapping results (Fig. S5) indicate that the changes of fluorescence intensities of reference areas during FRAP experiments (Fig. S6) were caused by photobleaching and exchange of the bleached TMR-Halo-FGFs from the bleaching area, rather than dissociation of Halo-FGF into the bulk culture medium. The imaging videos are examples of FRAP experiments for Halo-FGF1, Halo-FGF2 and Halo-FGF6.

Figure S1: The brightfield images corresponding to the images in Fig 2. The images *a*, *b*, *c*, *d*, *e*, *f*, *g*, *h* and *i* are corresponding to images *a*, *b*, *c*, *d*, *e*, *f*, *g*, *h* and *i* in figure 2.

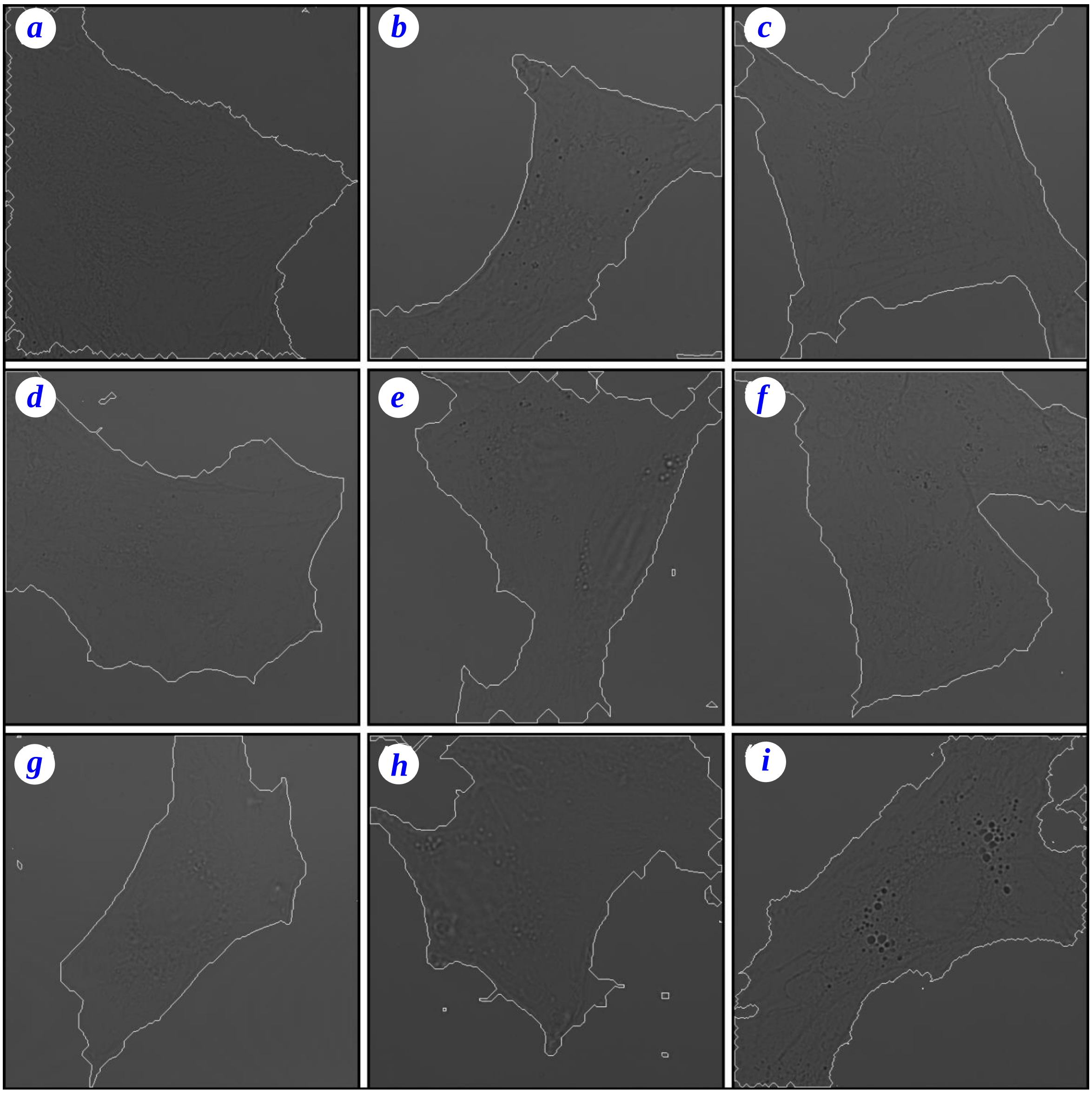

Figure S2: The brightfield images corresponding to the images in Fig 3. The images *a*, *b*, *c*, *d*, *e* and *f* are corresponding to images *a*, *b*, *c*, *d*, *e*, and *f* in figure 3.

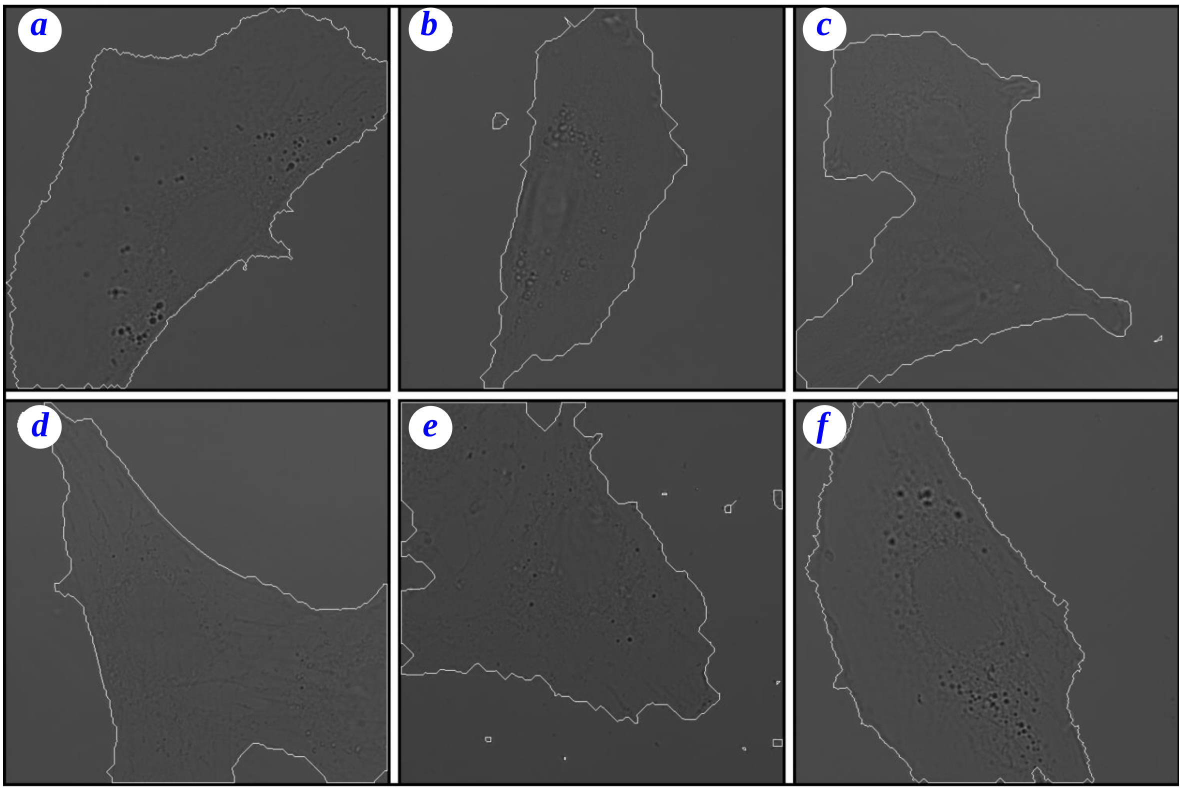

Figure S3: The brightfield images corresponding to the images in Fig 4. The images *a*, *b*, *c*, *d*, *e*, *f*, *g*, *h* and *i* are corresponding to images *a*, *b*, *c*, *d*, *e*, *f*, *g*, *h* and *i* in figure 4.

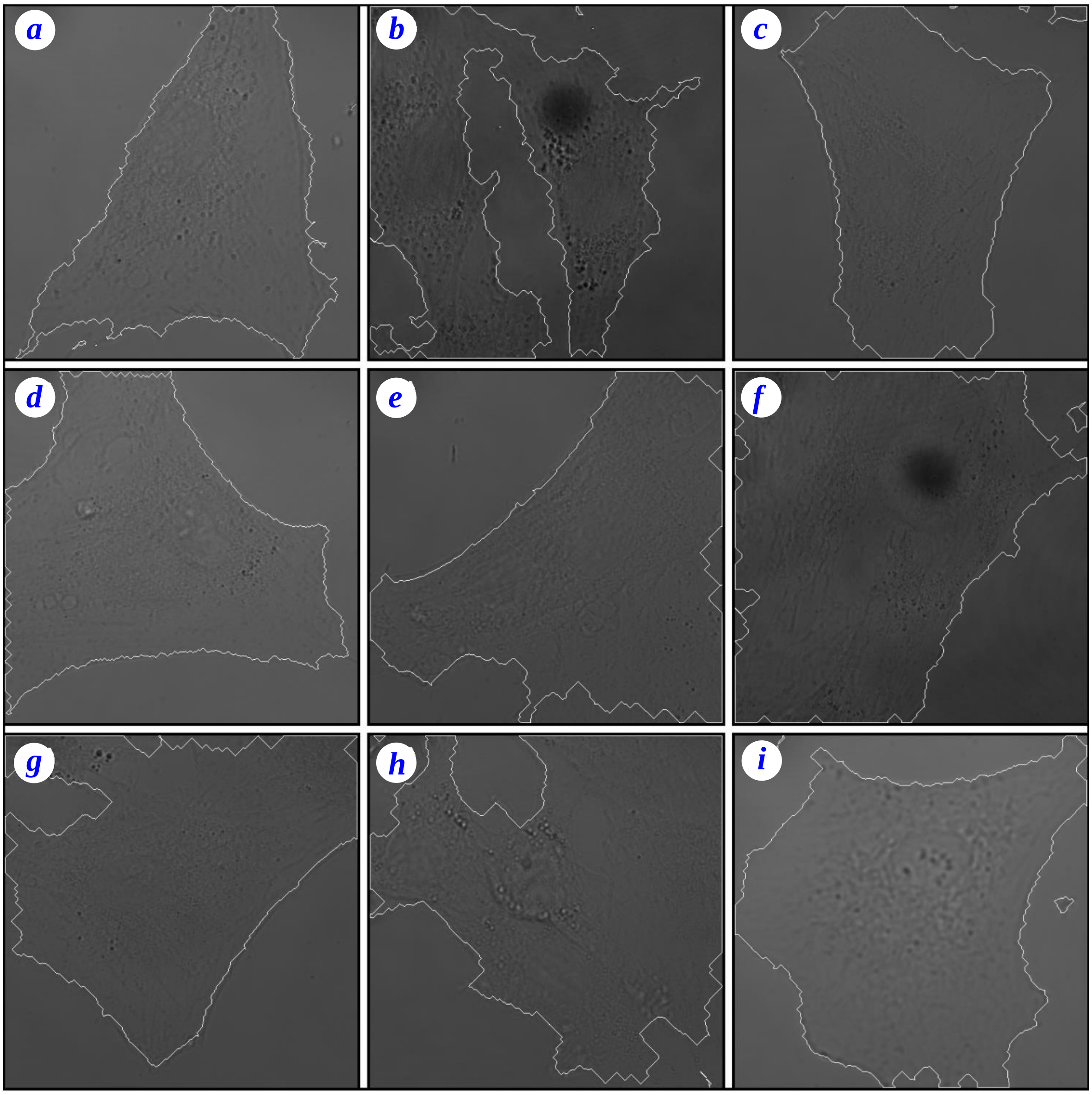

Figure S4: Binding of Halo-FGF20 and HaloTag to Rama 27 fibroblasts: (*a*): Rama 27 fibroblasts were incubated with 2 nM TMR-Halo-FGF20; **(*b*):** Rama 27 fibroblasts were incubated with 2 nM TMR-HaloTag. **(*c-d*):** The brightfield images channel for (*a*) and (*b*). Image size: 67.3 µm X 67.3 µm.

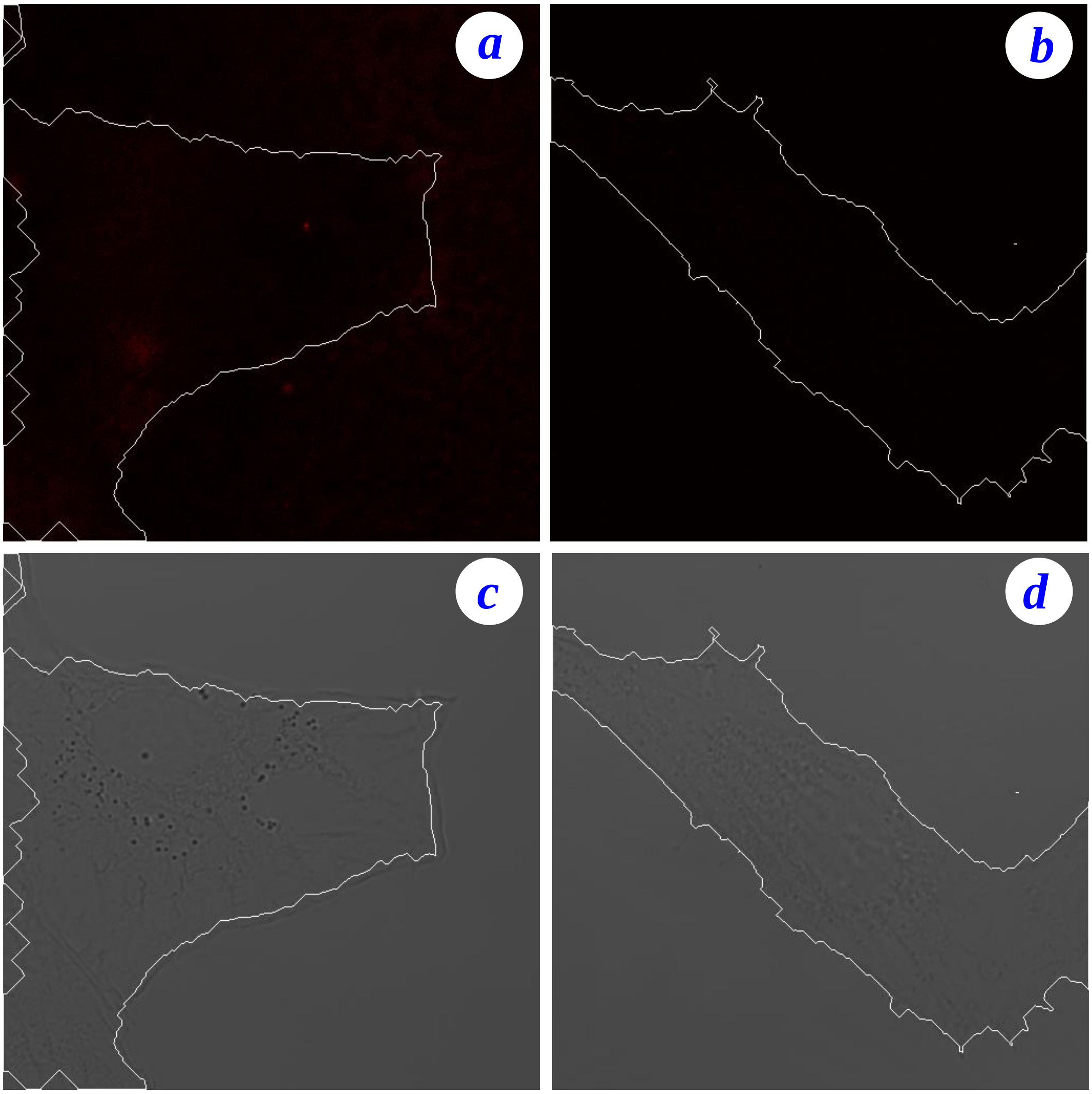

Figure S5: Dissociation of Halo-FGF1, Halo-FGF2 and Halo-FGF6 from Rama 27 pericellular matrix into the bulk culture medium. Rama 27 fibroblasts were incubated with 2 nM TMR-Halo-FGF1, TMR-Halo-FGF2 and TMR-Halo-FGF6. Ten images at 30 s interval were acquired for each area (six different areas for each Halo-FGF) to measure the dissociation of Halo-FGFs from the pericellular matrix into the bulk culture medium. The averaged fluorescence intensity was quantified as described in materials and methods. The fluorescence intensity lines above the dash line are the fluorescence intensities of Halo-FGF1. Halo-FGF2 is between the dash line and dotted line and Halo-FGF6 is below the dotted line.

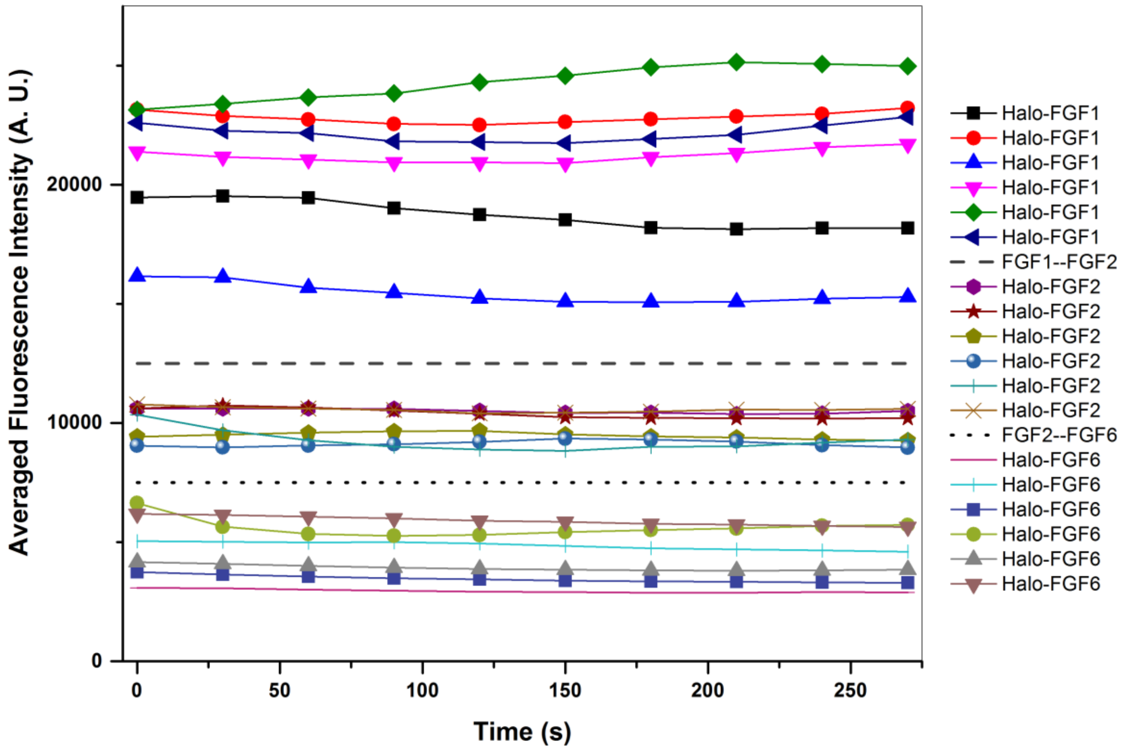

Figure S6: Photobleaching of TMR-Halo-FGFs during FRAP experiments. The fluorescence intensity of the reference area was extracted to determine the photobleaching of TMR-Halo-FGFs during the FRAP experiments. **(*a*):** Averaged fluorescence intensity of the reference area labelled with 2 nM Halo-FGF1. **(*b*):** Averaged fluorescence intensity of the reference area labelled with 1 nM Halo-FGF1. **(*c*):** Averaged fluorescence intensity of the reference area labelled with 2 nM Halo-FGF2. **(*d*):** Averaged fluorescence intensity of the reference area labelled with 2 nM Halo-FGF6.

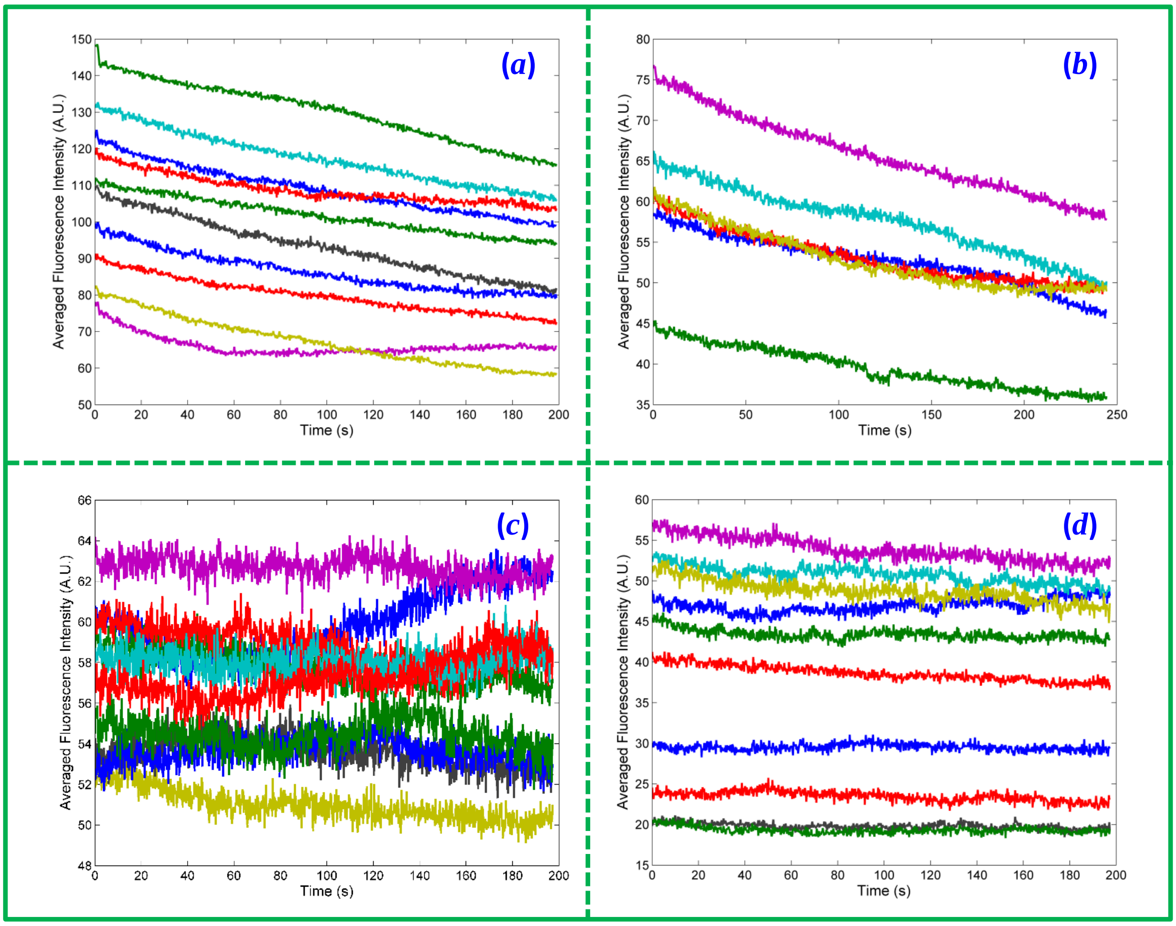

Figures S7-S10: FRAP movies for 2 nM Halo-FGF1 (S7), 1 nM Halo-FGF1 (S8), 2 nM Halo-FGF2 (S9) and 2 nM Halo-FGF6 (S10). Each video consists of 1000 images acquired over 197 s.

Table S1 Raw imaging data list in OMERO

The raw imaging data used in this article are stored on The Open Microscopy Environment (OMERO) and listed in the following website:

http://cci02.liv.ac.uk/gallery/show_project/1902/.

<table>
<tr><th>Folders</th><th>Included imaging data</th><th>Name note</th></tr>
<tr><td>Binding and control for FGF1</td><td>The binding images of Halo-FGF1 and the competitions and the heparan sulfate and chondroitin sulfate and dermatan sulfate digestions in Fig. 3 legend.</td><td rowspan="5">The name of each image contains three parts:

Protein name: HTF1/2/6/10/20 are Halo-FGF1/2/6/10/20;

Conditions:

Binding: 2 nM FGF binding to Rama 27 cells

8 uM FGF1/2 (HTF1/2): binding with 8 µM unlabelled FGF1/2 or Halo-FGF1/2

Heparin: binding in the presence of 4 µg/mL heparin

Hepnase: binding to Rama 27 fibroblast pericellular matrix digested with heparinase I, II and III

CSnase: binding to Rama 27 fibroblast pericellular matrix digested with chondroitinase ABC

Image number order: 01, 02 etc.</td></tr>
<tr><td>Binding and control for FGF2</td><td>As above for Halo-FGF2 in Fig. 2 legend.</td></tr>
<tr><td>Binding and control for FGF6</td><td rowspan="2">The binding images of Halo-FGF6 and Halo-FGF10 and the competitions and the heparan sulfate and chondroitin sulfate and dermatan sulfate digestions in Fig. 4 legend.</td></tr>
<tr><td>Binding and control for FGF10</td></tr>
<tr><td>Binding and control for FGF20</td><td>The binding images of Halo-FGF20 in Fig S4.</td></tr>
</table>

FRAP for FGF1	FRAP imaging data for Halo-FGF1	File names with ‘HTF1 AW 1nM’ are the FRAP imaging data for 1 nM Halo-FGF1 and the other files are the FRAP imaging data for 2 nM Halo-FGF1.
FRAP for FGF2	FRAP imaging data for Halo-FGF2	All the files are FRAP imaging data for 2 nM each Halo-FGF in the corresponding folder.
FRAP for FGF6	FRAP imaging data for Halo-FGF6	
FRAP for FGF10	FRAP imaging data for Halo-FGF10	
General control	Imaging data of the blank cell and cells incubated with Halo-TMR fluorescence dye or TMR-HaloTag	Rama27 cells: imaging data of blank Rama 27 fibroblasts;<br>TMR: imaging data of Rama 27 fibroblasts incubated TMR fluorescence dye;<br>HTG: imaging data of Rama 27 fibroblasts incubated TMR-HaloTag
Trapping data	The trapping imaging data for Halo-FGF1, Halo-FGF2 and Halo-FGF6	No special
